# Rapid Measurement of Soybean Seed Viability Using Kernel-Based Multispectral Image Analysis

**DOI:** 10.3390/s19020271

**Published:** 2019-01-11

**Authors:** Insuck Baek, Dewi Kusumaningrum, Lalit Mohan Kandpal, Santosh Lohumi, Changyeun Mo, Moon S. Kim, Byoung-Kwan Cho

**Affiliations:** 1 Department of Mechanical Engineering, University of Maryland, Baltimore County, 1000 Hilltop Circle, Baltimore, MD 21250, USA; insuck.baek@gmail.com; 2 USDA-ARS Environmental Microbial and Food Safety Laboratory, Henry A. Wallace Beltsville Agricultural Research Center, Beltsville, MD 20705, USA; Moon.Kim@ars.usda.gov; 3 Department of Biosystems Machinery Engineering, College of Agricultural and Life Science, Chungnam National University, 99 Daehak-ro, Yuseong-gu, Daejeon 34134, Korea; dewi.kusumaningrumm@gmail.com (D.K.); lalitm85@gmail.com (L.M.K.); santosh.sanny123@gmail.com (S.L.); 4 National Institute of Agricultural Sciences, Rural Development Administration, 310 Nonsaengmyeong-ro, Wansan-gu, Jeonju-si, Jeollabuk-do 54875, Korea; cymo76@gmail.com

**Keywords:** seed viability, near-infrared, multispectral imaging, variable importance in projection, kernel-based classification

## Abstract

Viability is an important quality factor influencing seed germination and crop yield. Current seed-viability testing methods rely on conventional manual inspections, which use destructive, labor-intensive and time-consuming measurements. The aim of this study is to distinguish between viable and nonviable soybean seeds, using a near-infrared (NIR) hyperspectral imaging (HSI) technique in a rapid and nondestructive manner. The data extracted from the NIR–HSI of viable and nonviable soybean seeds were analyzed using a partial least-squares discrimination analysis (PLS-DA) technique for classifying the viable and nonviable soybean seeds. Variable importance in projection (VIP) was used as a waveband selection method to develop a multispectral imaging model. Initially, the spectral profile of each pixel in the soybean seed images was subjected to PLS-DA analysis, which yielded a reasonable classification accuracy; however, the pixel-based classification method was not successful for high accuracy detection for nonviable seeds. Another viability detection method was then investigated: a kernel image threshold method with an optimum-detection-rate strategy. The kernel-based classification of seeds showed over 95% accuracy even when using only seven optimal wavebands selected through VIP. The results show that the proposed multispectral NIR imaging method is an effective and accurate nondestructive technique for the discrimination of soybean seed viability.

## 1. Introduction

Soybean is a major agricultural commodity in world trade, and is a rich source of protein and oil for consumption by both humans and animals. The latest data from the United States Department of Agriculture report that US soybean production increased by 59% between 2000 and 2017 [1]. In 2016, the total global production of soybean was approximately 335 million tons [2]. Although soybeans are produced by only a few countries, they are traded widely to meet soybean demand in every country in the world. More than 90% of global soybean production comes from the US, Brazil, Paraguay, and Argentina, while the biggest importers are China, Korea, and Japan [3]. Ensuring the quality of seeds is important for improving agricultural production and fulfilling the high demand for soybeans worldwide. A high quality soybean seed is defined as a varietally pure seed that is characterized by high viability and vigor; proper moisture content, size and weight; and is free from disease and disease organisms [4]. Viability measurements reflecting the likelihood that seeds will successfully germinate and develop into new plants under appropriate conditions is crucial to industrial field production [5]. Therefore, viability is considered the most important parameter of seed quality. Seed viability declines as seeds age, slowly at first but more rapidly with increasing time. Therefore, seeds with high viability can be stored safely for longer periods, and factors affecting seed viability have been studied intensively for many years.

Many different methods are available for determining seed viability, such as the germination test, tetrazolium-based test, biochemical test, and human inspection. However, these methods have several disadvantages. They are not only sample-destructive and labor-intensive, but also require complicated and time-consuming procedures to be performed by personnel with specialized training. Nondestructive methods for determining seed viability are thus highly sought after by the seed industry [6]. Among available nondestructive measurement methods, hyperspectral imaging (HSI) is a very promising tool for rapid determination of seed viability. The HSI technique in the near-infrared (NIR) range has been implemented successfully to evaluate various seed quality attributes [7], such as protein content determination in oilseeds, contaminant detection in wheat [8], identification of fungus-damaged kernels [9], identification of different seed classes based on moisture levels [10], quality measurement of aged seeds [11], hardness detection in maize [12], and viability measurement of corn and muskmelon seeds [13,14]. In the NIR region between 780 and 2500 nm, chemical bonds such as C–H, O–H, and N–H have high vibrational-frequency absorption, which reflects the strengths of the chemical bonds involved [15]. Since a large number of samples can be scanned simultaneously by HSI, the technique can overcome the limitations of conventional spectroscopy techniques that usually measure individual samples separately. HSI technology provides both spectral and image features for objects measured in bulk, and the 3D data hypercubes created by HSI allow for complete and reliable analysis of the intrinsic properties and morphological characteristics of the scanned object [14]. With the development of chemometric analysis techniques, NIR–HSI has emerged as a powerful analytical technique that combines spectroscopic analysis with spectral images for the rapid quality measurement of food and agriculture products.

Chemometric analysis is an inseparable part of spectroscopy-based multivariate data analysis. It contributes to the extraction of the useful information present in the spectra, while separating out noise or unwanted spectral outliers, thus facilitating data analysis for classification or prediction purposes [16]. The general steps involved in hyperspectral data processing are image processing, spectral preprocessing, variable selection, and multivariate chemometric analysis. Although variable selection is a very important step that can improve model performance, it may eliminate some useful information from the model. Moreover, using a small number of variables for prediction increases the influence of each variable on the final model [17]. Comparisons have been conducted for some variable selection methods in combination with multivariate analysis of hyperspectral data [14].

Although several studies have tried to use HSI technique on seed viability and quality measurements, no research has reported the use of an HSI technique combined with image processing and the optimum-detection-rate technique for the determination of soybean seed viability, to the best of our knowledge. This study investigates the feasibility of the HSI technique in combination with the PLS-DA, optimal variable selection method, and image processing technique, for determining viable and nonviable soybean seeds.

## 2. Methods

### 2.1. Sample Preparation

A total of 400 soybean seeds (*Glycine max* (L.) Merill) were purchased from the Korean bean sprout association. Two hundred of these seeds were untreated and used for the viable seed group in this experiment. The other 200 seeds were artificially aged: they were packed in plastic bags and incubated for nine days in a water bath maintained at 42 °C to accelerate seed respiration. After nine days, both of the artificially aged and untreated seeds were maintained in an incubator at 20 °C and 65% relative humidity to equilibrate the conditions.

Artificially accelerated aging in this manner can reduce seed germination capability without harming other seed qualities. The high temperature, between 42 and 45 °C, usually causes hormonal and metabolic inactivation [18]. A previous study showed that the seed germination rate after accelerated aging was similar to that of seeds stored for 18 months under conventional storage conditions [19]. This fact indicates that the accelerated aging of soybean seeds could affect the hydrolysis of proteins, lipids, and carbohydrates [20]. For this experiment of soybean seeds, the viable and nonviable soybean seeds appeared the same; i.e., no difference was observed in their color or other physical parameters.

### 2.2. SWIR Hyperspectral Imaging System

**Figure 1:**
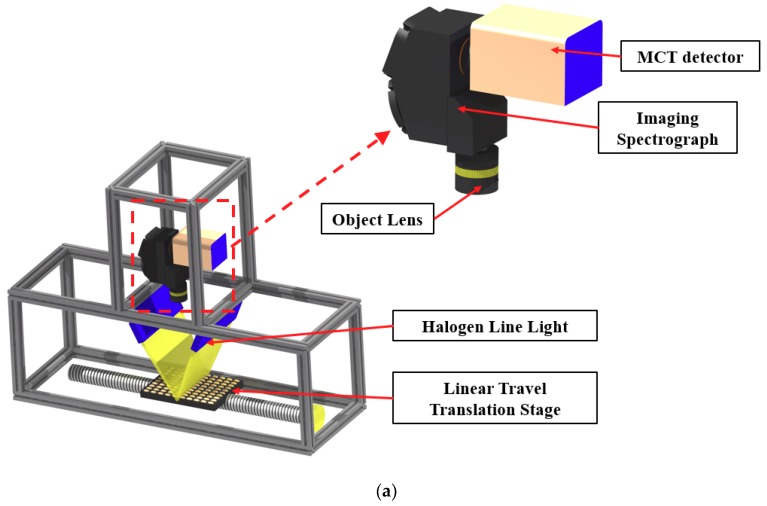

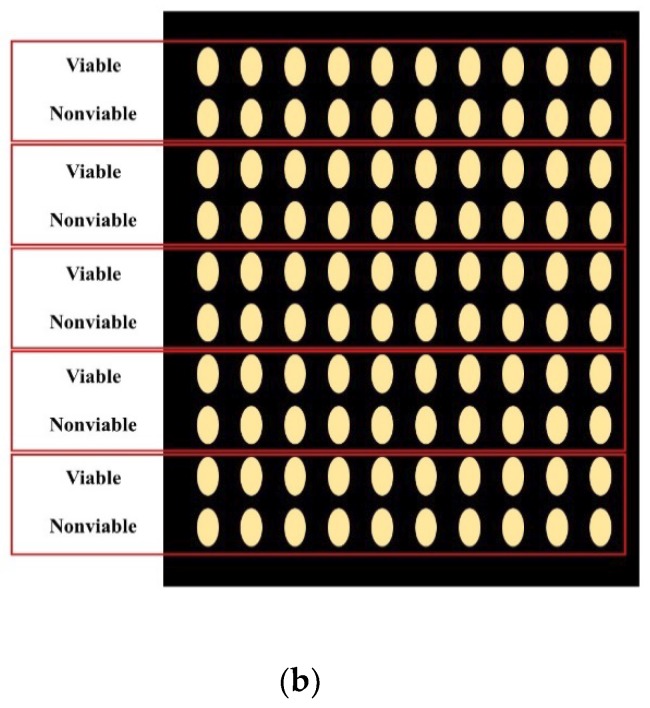
(**a**) Schematic representation of the SWIR-HSI system; (**b**) arrangement of soybean seeds on sample holder plate for HSI scanning.

A laboratory-based line-scan SWIR–HSI system (shown in Figure 1a) was used for collecting hyperspectral images of the soybean seeds. The system was composed of a line-scan spectrograph (SWIR, Headwall Photonics, Fitchburg, MA, USA) with a spectral range of 1000–2500 nm, a mercury cadmium telluride (MCT) detector (Model: Xeva-2.5-320; Xenics, Heverlee, Belgium), an imaging camera with 320 (spatial) × 256 (spectral) pixel resolution, a 25 µm slit, an objective lens (focal length 25 mm f/1.4), a motorized positioning table (Xslide, Velmex INC., Bloomfield, NY, USA) to move the samples across the camera’s field of view, a DC motor to control the speed of the conveying unit, and a halogen-tungsten line-light source (100 W × 6 lamps) connected to optical fibers for illuminating the samples during measurement. The data acquisition software was developed using Microsoft Visual Basic (version 6.0) on a Windows platform. Before the HSI scanning of the soybean samples, system parameters were adjusted to the following settings: 30 ms camera exposure time, −73.15 °C (200 K) detector cooling temperature, and 0.2 mm/scan sample increment. The spectra were calibrated using a general-purpose cool-white fluorescent lamp, which emitted wavelengths for mercury, terbium, europium and argon. The white Teflon flatted panel reflected peak wavelengths via illumination with the cool-white fluorescent lamp, and the channel numbers of the spectral axis in the hyperspectral cube corresponded to known wavelength peaks by linear regression.

### 2.3. Image Acquisition and Correction

A 100-seed sample holder plate was used to hold seeds arranged in a 10 × 10 grid for imaging the seed samples from both sample groups, alternating between rows of viable seeds and nonviable seeds (Figure 1b). Placed on the positioning table that was controlled by stepping motor, the seed sample plate was scanned line-by-line using the HSI system. The acquired SWIR hyperspectral images of the seed samples were stored in a three-dimensional (3D) format called the 3D hypercube, consisting of two spatial dimensions (*x* and *y*), and one spectral dimension (*λ*). White reference and dark current images were acquired to calculate reflectance values. The dark current image (0% reflectance) was acquired by covering the camera lens, while the white image (~99% reflectance) was acquired by using a white teflon sheet. Calibrated hyperspectral reflectance images of the samples were calculated by applying the following equation:

(1) $$I_{R}=\frac{\left(I_{o}-I_{d}\right)}{\left(I_{w}-I_{d}\right)},$$

where, *I_R_*, *I_o_*, *I_d_* and *I_w_* were the calibrated image, original image, dark current image, and white reference image, respectively.

### 2.4. Data Extraction and Preprocessing

**Figure 2:**
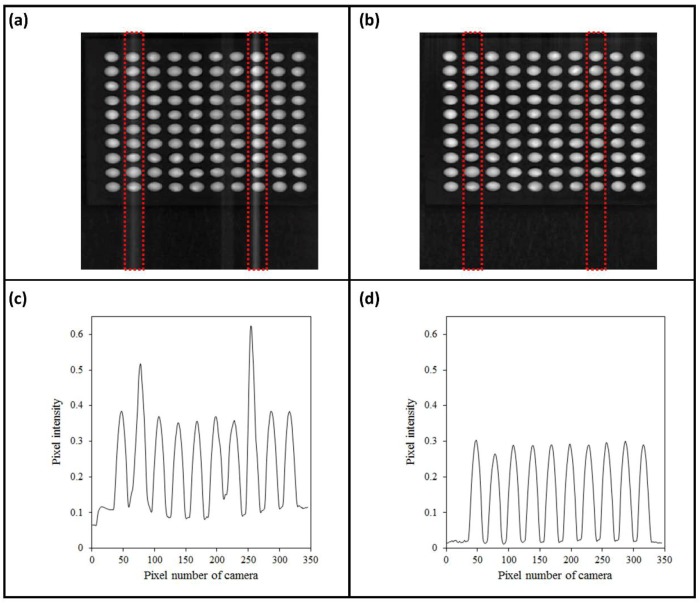
Hyperspectral band images of soybean seeds: (**a**) before and (**b**) after baseline correction and (**c**,**d**) showing average of corresponding pixel intensity.

The calibrated hyperspectral image tends to be corrupted by unstable light scattering, which results in a baseline shift (Figure 2a). To avoid this affect, a baseline correction method was applied to improve the quality of the image and spectrum of the seed sample. Figure 2b was constructed to present a comparison between the unclear image and clear image at 1365 nm.

After the baseline correction, region-of-interest (ROI) selection was performed to extract the spectral signatures from the seed samples. The binary mask image made by a simple threshold method for the 1300 nm image was used to discriminate the seed areas from background among the hyperspectral images. The spectral information of the seed samples could be extracted from the ROI of the masked image. In addition, the ROI spectral data of the seed samples were subjected to various preprocessing techniques including normalization (mean, range, and max normalization), SNV calculation, and smoothing. These pretreatment techniques were utilized to improve the spectral data by removing irrelevant information and retaining valuable spectra for providing better performance from the multivariate classification model that was developed in this study.

### 2.5. Partial Least-Squares Discriminant Analysis (PLS-DA)

In this study, a PLS-DA model was built to discriminate between the viable and nonviable soybean seeds. PLS-DA is a supervised classification analysis technique that classifies a new group of samples into predefined known classes according to their measured features [21]. This analysis method has been previously applied for the assessment of various seed quality attributes, and has been demonstrated to be a powerful and accurate method for classification [22]. The partial least-squares regression (PLS-R) analysis is well suited for HSI data where the data are composed of more variables than observations with high correlation. A detailed description of the basic theory of PLS-DA was omitted for brevity and can be found in many articles [14,23].

For construction of the PLS-DA model, the entire preprocessed full pixel-based spectral data set from viable and nonviable seeds were arranged in the independent variable matrix, while the dependent variable matrix was categorical and contained artificial values of 0 or 1, corresponding to the seed category (“0” for nonviable seeds and “1” for viable seeds). For the classification of each seed category to its assigned value, a threshold value of 0.5 was set between both groups to classify the two groups. Moreover, the entire data (spectral data) obtained from 400 seed samples were split into two subsets: calibration set (containing 75% of the total data) and validation set (containing 25% of the total data). The calibration set was used for developing the model and the validation set was used for evaluating the actual predictive ability of the developed model.

### 2.6. Variable Importance in Projection (VIP)

The vast amount of spectral data generated by HSI, exhibiting high covariance and containing a considerable amount of redundant information, often requires large amounts of storage space and computation time for data processing. The objective of the variable-selection method is to select optimum variables that are composed of important information for improving validation performance, and to eliminate unwanted information from the spectral data, thus reducing computation time [24]. The VIP variable-selection method is commonly used to estimate the importance of the *X* variables in the multivariate models based on projections to latent structures, i.e., PLS method [17]. In general, a VIP score value below one identifies an unimportant variable which probably will be eliminated while reducing the data volume [17]. Moreover, even if a wavelength has a VIP score value above one, the contiguous wavelengths in the data set can lead to problems of multi-collinearity and information redundancy, since contiguous wavelengths connote similar spectral information [25]. The number of variables can be reduced by selecting major peaks with VIP scores above one and, eventually, developing the PLS-DA model using those selected wavebands.

### 2.7. Image Processing

**Figure 3:**
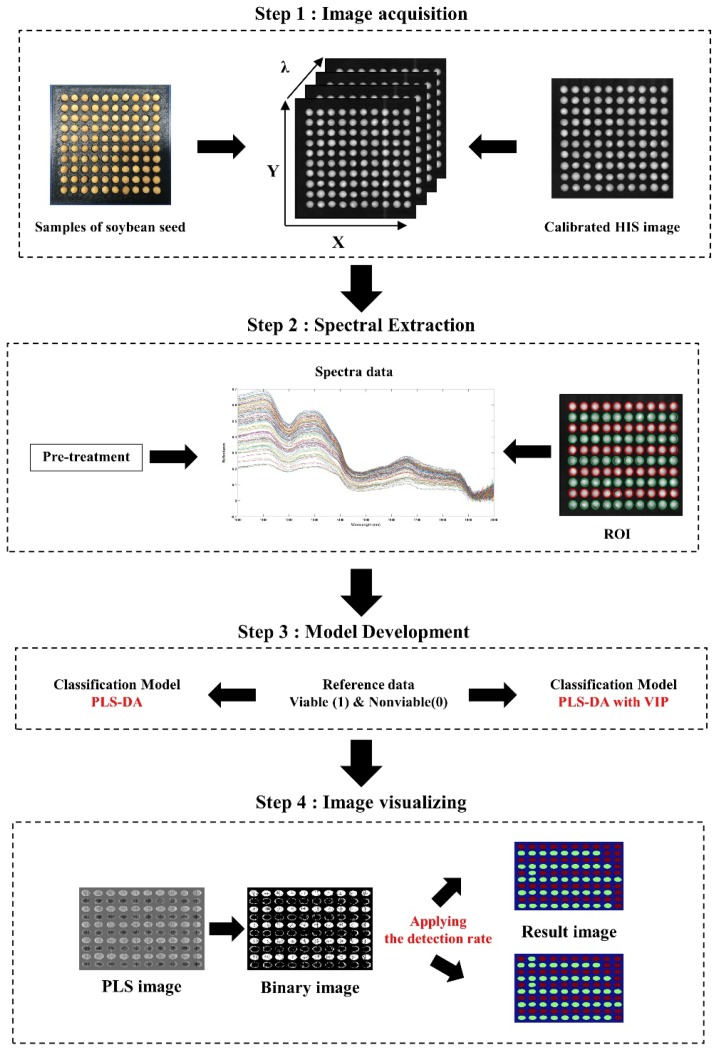
HSI data processing workflow used for viability determination of soybean seed samples.

One of the unique abilities of using HSI in combination with chemometric analysis is the visualization of the spatial profiles of samples based on their chemical compositions, also known as the chemical image. In this study, this was used as an alternative strategy for testing the PLS-DA model efficiency for the discrimination of viable and nonviable soybean seeds. The visualization images of the seed samples are generated by multiplying the coefficient values (obtained from the PLS-DA model) with each pixel of the preprocessed hyperspectral image. Before developing the visualization image (PLS-DA image), the background is eliminated by applying a simple threshold method. Then, the PLS-DA image without background is converted to a binary image using a 0.5 threshold value (since viable seeds were modeled as “1” and nonviable seeds as “0” during model development). In the binary image, the number of pixels with intensities above 0.5 are counted for each seed. Finally, the seed is classified as viable or nonviable, depending on the detection rate, using Equations (2) and (3). For example, one seed consisted of 100 pixels after applying the PLS-DA model and the detection rate in use was 50%. The number of pixels exhibiting an intensity value over 0.5 were counted. If the number of pixels (with intensity >0.5) was greater than 50% of the total number of pixels, the seed was considered viable and was displayed in red. If the number of pixels counted was less than 50% of the total number of pixels, the seed was considered nonviable and was displayed in green in the final image. Figure 3 details the steps used for processing the soybean data. To calculate the optimum detection rate, we used a receiver operating characteristic (ROC) curve. This curve presents relative trade-offs between the true-positive rate (called sensitivity) and false-positive rate (called specificity), where X and Y axis indicates specificity and sensitivity, respectively. The perfect trad-off value would result in a point in the upper left corner in the ROC space, representing 100% classification accuracy. Therefore, the ROC curve depicts the performance of a model by using the entire range of classification trade-off values from 0 to 100 in this study. Further detail information and interpretation of ROC curves was described elsewhere in the literature [26,27]. In this study, all programming was implemented in MATLAB 2012b software (MathWorks, Natick, MA, USA) using the PLS and the image-processing toolboxes.

(2) $$\mathrm{viable}\;\mathrm{seed}=\frac{\mathrm{number}\;\mathrm{of}\;\mathrm{detected}\;\mathrm{pixels}\;}{\mathrm{number}\;\mathrm{of}\;\mathrm{total}\;\mathrm{pixels}\;\mathrm{in}\;\mathrm{seed}}\times 100\geq detection\;rate$$

(3) $$\mathrm{nonviable}\;\mathrm{seed}=\frac{\mathrm{number}\;\mathrm{of}\;\mathrm{detected}\;\mathrm{pixels}\;}{\mathrm{number}\;\mathrm{of}\;\mathrm{total}\;\mathrm{pixels}\;\mathrm{in}\;\mathrm{seed}}\times 100<detection\;rate\;,$$

where the detected pixels are the pixels in PLS-DA image with intensities higher than the threshold value (0.5).

### 2.8. Germination Test

For validation, a germination test was conducted on the seed samples using a paper-based method, following International Seed Testing Association (ISTA) rules. One hundred seeds each of viable and nonviable soybean were placed on moist paper, and then stored in an incubator at 25 °C and 65% relative humidity without light. After nine days, seeds that had produced seedlings with shoots longer than 1 cm were counted as viable seeds. The germination rate of normal seeds was 98%, while that of the artificially aged seeds was 0%.

## 3. Results and Discussion

### 3.1. Spectral Characteristics of Soybean Seeds

**Figure 4:**
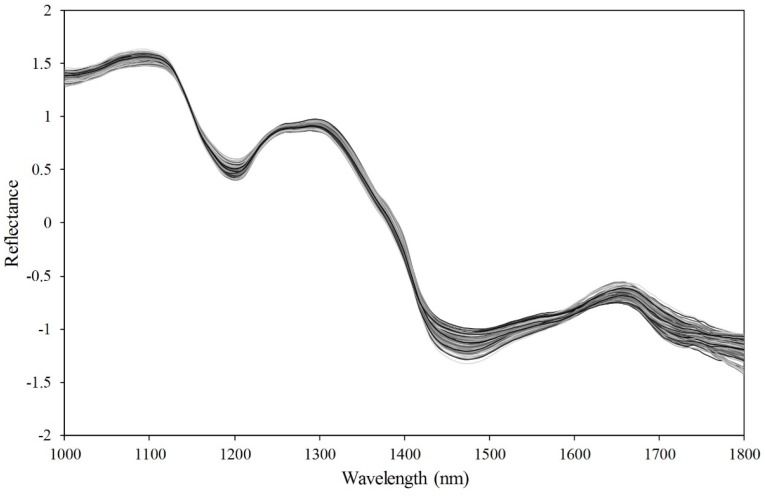
Average spectra of each soybean seeds in 1000–1800 nm.

The average SNV-pretreated spectra of the nonviable and viable soybean seeds are shown in Figure 4. In general, the SNV pretreatment method removes data noise such as that from light scattering, morphological differences, and sensor sensitivity. Figure 4 presents the general peaks and valleys associated with the chemical properties of the soybean seeds. For example, the peaks around 1300 nm and 1600 nm are associated with fiber content, and the valleys around 1200 nm and 1400 nm represent proteins and oils in the seed [9,14]. However, the differences were not distinctive between viable and nonviable soybean seeds. This result indicated that the simple bands methods such as band ratio or simple thresholding methods using one bands cannot be used for discrimination of this study. In addition, multivariable methods are needed since the entire spectrum pattern between viable and nonviable soybean seeds is similar. Spectral data at wavebands over 1800 nm were omitted because no significant information about the seeds were present in the noise pattern. The final model was developed using wavebands between 1000 and 1800 nm.

### 3.2. PLS-DA Classification Using Entire Wavelengths

PLS-DA was used to build a classification model for viable and nonviable soybean seeds. Viable and nonviable seeds were divided into calibration and validation sets. Table 1 describes the latent variables and RMSECV captured by the PLS-DA model for the various preprocessing methods. Using a large number of latent variables provides superior performance in fitting of the calibration and validation data; however, this can lead to over-fitting of the model. To overcome this problem, a 100-fold cross validation method to choose the optimal number of latent variables was employed. All of the models flatten out after around 20 latent variable numbers in the RMSECV curve. Thus, the optimum latent variable number corresponding to the minimum value in the RMSECV curve were chosen. This study did not use full cross validation (commonly used in other studies), because the 150,000 spectra available was more than sufficient for modeling, and modeling with full cross validation using so much data would be a very time-consuming process. The resultant classification accuracies obtained from the PLS-DA model using various preprocessing methods are summarized in Table 2. It can be seen that all the preprocessing methods performed well and attained similar classification accuracies for viable and nonviable seed samples in the calibration set, while the nonviable seeds were predicted with higher accuracies (>94%) than the viable seeds (<90%) for the validation set. This probably resulted from the seed aging treatment for the nonviable group, since all nonviable (treated) seeds exhibited similar spectral features and their seed moisture concentration had been equilibrated. Accuracy may also have been decreased due to the calculation based on classification of each pixel, where viable seeds with more than 50% misclassified pixels were considered as nonviable seeds even though they still had viability.

**Figure 5:**
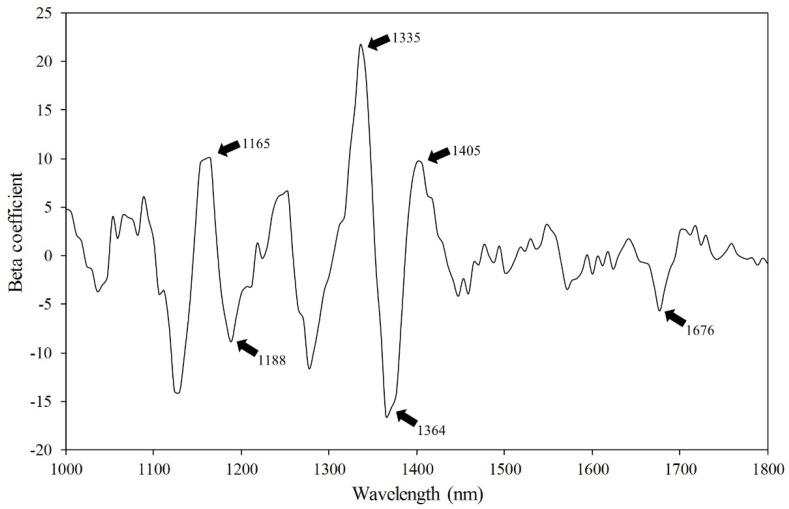
Beta coefficient of the PLS-DA model using the original (raw) full wavelengths (B_0_ = 0.4533).

The beta coefficients plot (Figure 5) from the PLS-DA was used to identify wavelengths carrying useful information about the chemical features of the soybean seeds. The beta coefficient value measures how strongly each predictor variable influences the dependent variable. The beta coefficient, also known as the standardized coefficient, was used to interpret the direction of the relationship between spectrum as predictor variables and viability as the criterion variable. The peaks and valleys of the beta coefficient curve showed some significant differences in energy absorption between viable and nonviable soybean seeds. The peak observed around 1165 nm has been associated with C–H (carbohydrate) second overtone stretching resulting from the CH_3_ functional group [13]. This peak was the major discriminating region in the model for the classification of the viable and nonviable seeds and may be closely associated with the germination ability of the soybean seed. The valley around 1364 nm has been reported to be related to the combination C–H stretching resulting from the absorption by CH_3_ [12]. The peak around 1405 nm is related to the O–H bonds of oil, and could be significantly related to the condition of soybean viability [28,29]. Fiber and starch contents associated with wavebands at 1188 and 1335 nm related to combination C–H stretching [9,30,31,32]. Damaged soybean seeds have been found to have a lower fiber content than sound soybean seeds [33]. The absorption band around 1676 nm was related to the first overtone of the C–H stretching vibration of the methyl and methylene groups [14]. Some valleys and peaks in the beta-coefficient plot could be used to interpret the interactions of oil, fiber, and starch content, in terms of the viability of soybean seeds.

### 3.3. PLS-DA Classification Using VIP Selected Variables

**Figure 6:**
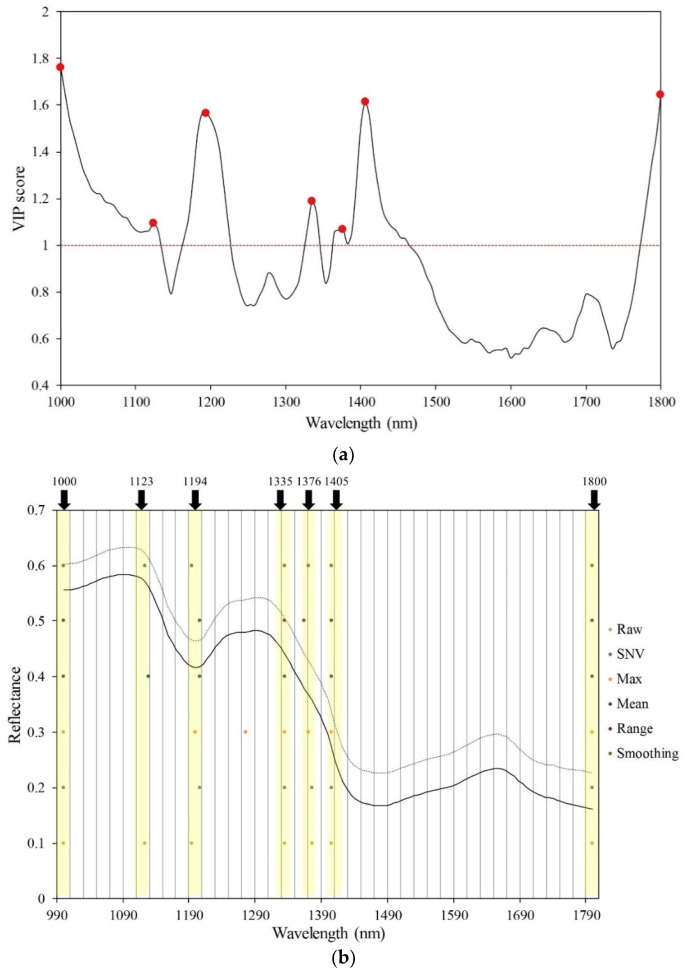
(**a**) VIP score plot indicating the key wavelengths selected for model development with original raw spectrum and (**b**) indicate average spectrum data and selected wavelength with each pretreatment methods (yellow bar is common key wavelength).

These peaks in the VIP score plot calculated from the PLS-DA model provide important information about variables that may be related to organic components and those responsible for the germination ability of the soybean seed. From the VIP scores, variables (wavebands) that are important for the projection of the PLS-DA model can be easily observed. Figure 6a shows VIP scores marked at key wavelengths and Figure 6b (the yellow bars) shows the key wavelengths for discrimination of viable and nonviable soybean seeds and the dot mark in the wavebands used in each preprocessing. Commonly, the wavelengths chosen based on VIP scores are 1000, 1123, 1194, 1335, 1376, 1405, and 1800 nm, which are known to be related to changes in chemical composition such as protein, fatty acid and starch that can be strong indicators for loss of seed viability. Due to the hydrolysis of proteins and fats during seed aging, nonviable seeds tend to have increased concentrations of free fatty acid, acid phosphate, and amino acids [34]. The fatty acid and protein contents are important for viability of oil seeds in general, which include soybean seeds.

The several variables selected using the VIP method were used in the PLS-DA model for the classification of viable and nonviable seeds. The classification results from the selected variables are presented in Table 3. Overall, the performance of the PLS-DA model developed with several wavelengths is slightly lower than that of the PLS-DA model developed with full wavebands (Table 2). However, the accuracy calculated from using the pixel-based spectra is not critical because the optimal detection rate should be obtained from the hyperspectral image instead of the pixel-based spectra. Thus, the kernel-based classification results of the two PLS-DA models using several wavelengths using the full spectra were provided by applying the optimal detection rate for classifying the two groups. These methods can reduce the number of variables and show the important variables that influence the viability of soybean seeds. Variable selection will be very important for future real-time online application of hyperspectral measurements for seed viability. Using the VIP-based variable-selection method, future imaging system will be cheaper and less time-consuming and optimum wavelengths will be beginning steps for application in multispectral devices for online measurements. In real-world applications, the multispectral imaging technique is commonly used; it has a lower waveband range and is faster and cheaper than the hyperspectral device.

### 3.4. Kernel-Based Classification of Viable and Nonviable Soybean Seeds

The pixel-based classification results from the PLS-DA model developed with whole variables and VIP-selected variables are compared and shown in Table 2 and Table 3. From the tables, it is observed that both the calibration and validation sets attain similar accuracies; however, the PLS-DA model developed with the VIP-selected variables yields slightly lower accuracy than that of the one developed with whole variables. This could be a consequence of using a smaller number of variables, which introduces comparatively lower variability to the model. In addition, in all cases, higher classification accuracy is achieved for the nonviable group of seeds, compared to the viable group, which may be the result of the aging treatment as mentioned earlier.

The classification models developed and discussed above are based solely on the spectral features of each pixel; however, seed viability is a concept related to the entire seed. Therefore, each pixel of the seed image cannot be considered to be representative of the viability status of the seed.

**Figure 7:**
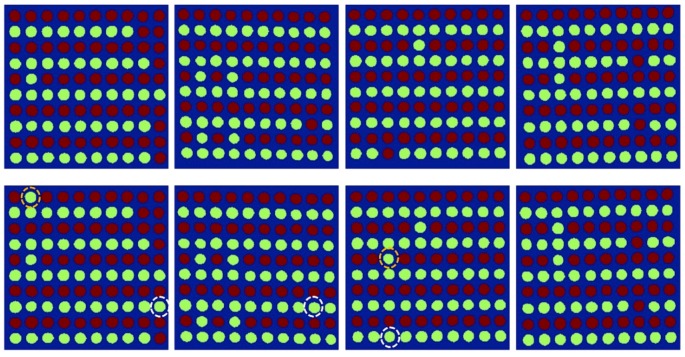
The resultant images by VIP PLS-DA model from raw data in the first row with 50% detection rate and in the second row with optimum detection rate. (Viable: red; non-viable: green.)

Therefore, eventually, a kernel-image-processing–based classification strategy is used to compensate for the pixel (spectral features)-based misclassification. Thus, each seed sample is classified as viable or nonviable, based on the numbers of pixels in the PLS-DA–based binary images correctly classified by calculating the optimal detection rate for each kernel seed image using the ROC curve. As a general practice, a 50% detection rate (threshold) is used to classify the two groups, similar to the 0.5 threshold value used in this study. However, the selection of an optimal detection rate will obviously improve accuracy. Hence, an optimum detection rate was calculated considering the lowest numbers of false-positive and false-negative classifications. Table 4 presents the AUC, optimum detection rate, and accuracy of each pretreatment method. Figure 7 shows the resultant images from the PLS-DA binary images, obtained using the detection rate of 50% and those generated using an optimal detection rate.

**Figure 8:**
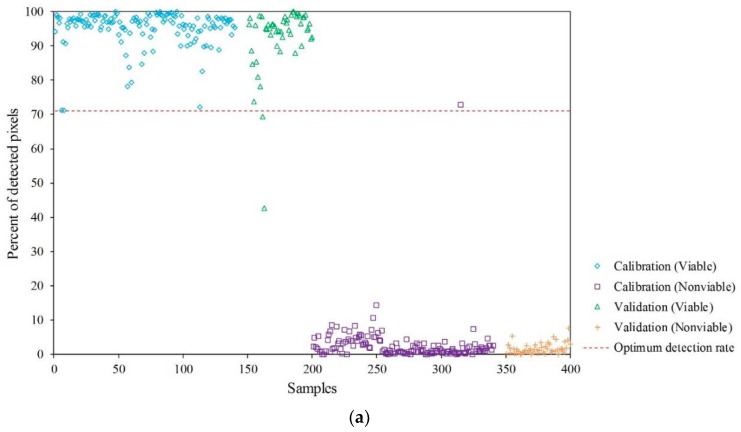

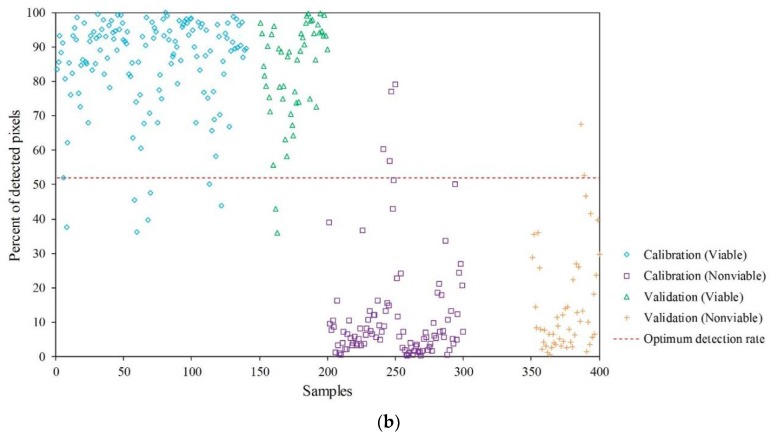
Graphical representation of results of image-based classification of viability of soybean seeds in the calibration and validation sets of whole spectral data (**a**) and VIP-selected variables (**b**) using optimum detection rate.

The final color-coded images for all four replications and two different detection rates (50% and optimal detection rates) for discrimination between viable and nonviable soybean seeds, based on the PLS-DA–VIP model, are shown in Figure 8. There is a notable difference (marked with dashed circles) in the classification results as three more nonviable seeds have been classified correctly using the optimal detection rate; however, two additional viable seeds have been misclassified as being nonviable. This is because the total number of pixels of misclassified viable seeds in the binary image were >50% but <52% of the whole seed. Thus, they were counted correctly as viable when the 50% detection rate was used, but were misclassified when the optimal detection rate was used. However, the common practice of seed companies is to completely discard the nonviable seeds, irrespective of whether some viable seeds are discarded along with them or not. Therefore, a threshold value based on the optimal detection rate is considered effective for this particular application because it minimizes the number of false-positive (nonviable seeds but classified as viable) classifications.

The percent of pixels detected in the seed, based on image analysis, is given in Figure 8. As shown in Table 4, the results of the PLS-DA model developed with VIP-selected variables are comparable to those of the model developed with all the spectral variables. It is interesting to observe that the VIP-selected PLS-DA model shows considerably lower classification accuracy than the model developed with whole variables. However, the accuracy increases significantly when an optimum-detection-rate method is used along with kernel-based image processing strategy. In addition, from Table 4, it is observed that the results of the PLS-DA–VIP model with only seven variables can achieve a classification accuracy >95%, without the use of any data pretreatment method. Though the classification accuracy is slightly lower than that of the model developed with whole variables, the performance of the PLS-DA–VIP model is still acceptable because it was developed with a much smaller number of variables, which reduces the computational time and complexity. Usually it is not easy to find the optimum trade-off value with the resultant PLS-DA image to discriminate two groups as in the previous study [13] because the pixel value of PLS-DA image is sensitive to the classification model and drifted around 0 and 1. In addition, a representative spectrum of an individual seed is limited to determine the portion of damaged or nonviable areas in the sample, which could be a significant source of errors. The kernel-based image processing method overcomes the limitation by adopting the optimum trade-off value for the number of evaluated pixels of two groups in the seed kernel. Most importantly, using the selected wavebands, a multispectral camera can be constructed for the same purpose, which can be applied in the viability analysis of soybean seeds with the advantages of being economically reasonable and fast.

## 4. Conclusions

A SWIR hyperspectral imaging system was optimized and used for NIR-based discrimination of viable and nonviable soybean seeds. The system is advantageous for nondestructive viability measurement since it can handle bulk measurement and an automatic seed separation mechanism can be easily synchronized with it. A kernel-based image processing technique was adopted to classify the whole seed as viable or nonviable instead of classifying individual pixels of hyperspectral images. The experimental results of this study demonstrate that the PLS-DA–VIP model developed with only several wavebands can determine the viability of soybean seeds with high accuracy (>95%). Therefore, from a practical point of view, using the selected bands, a multispectral imaging system can be envisaged in the near future, which will offer the advantage of being fast and economically reasonable for the measurement of soybean viability.

## Figures and Tables

**Table 1:** Optimum number of latent variables and RMSECV explained by PLS-DA models with various preprocessing techniques.

Preprocessing	Latent Variables	RMSECV
Raw	14	0.312
SNV	12	0.304
Max	14	0.310
Mean	12	0.322
Range	14	0.303
Smoothing	17	0.312

**Table 2:** Calibration and validation results based on pixels (unit: %) of PLS-DA model developed with different preprocessing methods using full wavelengths.

Calibration (*n* = 149,884)	Raw	SNV	Max	Mean	Range	Smoothing
Viable	91.0	91.4	91.2	90.7	91.4	91.0
Non-viable	92.8	92.7	92.8	93.1	92.7	92.8
Total	91.9	92.1	92.0	91.9	92.1	91.9
**Validation (*n* = 50,336)**						
Viable	89.0	89.4	89.1	88.6	89.4	89.0
Non-viable	94.6	94.8	94.9	95.1	94.7	94.6
Total	91.8	92.1	92.0	91.8	92.1	91.8

**Table 3:** Calibration and validation results on pixels (in percent) of PLS-DA VIP model with selected wavelengths.

Calibration (*n* = 149,884)	Raw	SNV	Max	Mean	Range	Smoothing
Viable	82.8	87.1	83.2	84.9	80.2	85.9
Non-viable	84.7	88.8	86.5	89.2	79.5	88.1
Total	83.7	88.0	84.9	87.1	79.9	87.0
**Validation (*n* = 50,336)**						
Viable	80.8	84.1	80.0	81.0	76.7	85.6
Non-viable	82.7	91.1	88.3	91.0	81.8	87.3
Total	81.8	87.6	84.1	86.1	79.3	84.5

**Table 4:** Classification results for seed image using Optimum detection rates for each pretreatment method.

PLS-DA with Full Wavelengths	Optimum Detection Rate (%)	AUC	Calibration (*n* = 300)		Validation (*n* = 100)	
			Viable Accuracy (%)	Non-Viable Accuracy (%)	Viable Accuracy (%)	Non-Viable Accuracy (%)
Raw	71.3	0.9999	100	99.3	96.0	100
SNV	63.4	0.9999	100	99.3	98.0	100
Max	56.3	0.9999	100	99.3	98.0	100
Mean	49.4	0.9998	100	99.3	98.0	100
Range	63.0	0.9999	100	99.3	98.0	100
Smoothing	70.9	0.9999	100	99.3	98.0	100
**PLS-DA with VIP**						
Raw	52.0	0.9947	95.3	97.3	96.0	96.0
SNV	33.9	0.9992	100	98.0	96.0	98.0
Max	56.0	0.9941	94.7	97.3	96.0	98.0
Mean	43.1	0.9959	95.3	98.0	96.0	98.0
Range	49.7	0.9717	94.0	90.0	96.0	94.0
Smoothing	55.7	0.9996	99.3	98.7	98.0	100
